# Growing with dinosaurs: a review of dinosaur reproduction and ontogeny

**DOI:** 10.1098/rsbl.2024.0474

**Published:** 2025-01-15

**Authors:** Kimberley E. J. Chapelle, Christopher T. Griffin, Diego Pol

**Affiliations:** ^1^ Department of Anatomical Sciences, Stony Brook University, Stony Brook, NY, USA; ^2^ Evolutionary Studies Institute, University of the Witwatersrand, Braamfontein, Johannesburg, South Africa; ^3^ Department of Geosciences, Princeton University, Princeton, NJ, USA; ^4^ Museo Argentino de Ciencias Naturales Bernardino Rivadavia, Buenos Aires, Argentina; ^5^ Consejo Nacional de Investigaciones Cientificas y Tecnicas (CONICET), Buenos Aires, Argentina

**Keywords:** reproduction, growth, development, Dinosauria

## Abstract

Since the start of the twenty-first century, there has been a notable increase in annual publications focusing on dinosaur reproduction and ontogeny with researchers using these data to address a range of macroevolutionary questions about dinosaurs. Ontogeny, which is closely tied to osteological morphological variation, impacts several key research areas, such as taxonomic diversity, population dynamics, palaeoecology, macroevolution, as well as the physiological and reproductive factors driving ecological success. While these broad studies have significantly advanced our understanding of dinosaur evolution, they have also revealed important challenges and areas needing further investigation. In this review, we aim to outline some of these challenges in major research areas linked to dinosaur ontogeny, namely reproductive biology, osteohistological growth strategies, morphological osteological variation and the link between ontogeny and macroevolution. We also offer some recommendations for best practices and promising future research directions. These recommendations include increasing sample sizes through fieldwork and exhaustive use of pre-existing fossil collections, using micro-computed tomography (μCT) scanning methods to increase dataset sizes in a non-destructive manner, methodical collection and reposition of μCT scan data, assessing ontogenetic maturity, establishing consistency in terminology and methods and building comprehensive extant comparative datasets.

## 1. Introduction

Dinosaurs are an extremely successful lineage, having appeared 235 million years ago, survived two mass extinctions and today being represented by the most diverse group of land vertebrates. Several growth and reproductive factors play a role in a species’ ability to thrive, especially in post-extinction periods when reproductive pressure is increased. To study these, scientists can rely on several lines of evidence including eggshell structure and composition, egg arrangement, clutch size, nest numbers and arrangement, bone and tooth histology and fossil skeleton (both embryonic and adult) morphology, anatomical position and preservation [1].

Scientific interest in dinosaur ontogeny and reproductive biology began soon after the recognition of the group, with the first descriptions of dinosaur eggs and eggshells appearing in the literature as early as 1859 [2,3]. Although these were attributed to birds and crocodiles at the time, the word dinosaur had only been coined 17 years prior [4] and was not yet familiar. Remains of immature dinosaurs would take a few more decades to be identified. The first tentative juvenile dinosaurs reported in the literature were recognized as such due to their small size and incomplete ossification [2,5–10]; however, the first truly comprehensive studies of reproductive biology and ontogenetic changes in a dinosaur taxon came over a century later, with the discovery and naming of *Maiasaura peeblesorum* [11]. The holotype skull PU 22405 was found in proximity to the skeletons of 15 small individuals in a ‘nest-like’ structure. This kickstarted over 30 years of collecting efforts and led to a slew of ontogenetic studies for decades to come including eggshell structure, nesting behaviours, parental care, embryonic anatomy, growth dynamics, tooth development, ontogenetic morphological variation and locomotion [12–20], making *M. peeblesorum* a model organism for studying dinosaurian reproduction and development.

Since the turn of the millennium, the number of annual publications on immature dinosaurs has greatly increased [21], with researchers using ontogenetic evidence to untangle many other aspects of dinosaur macroevolution. Ontogeny is fundamentally linked to morphological variation, and has implications for a variety of phylogenetic and palaeobiological questions, including taxonomic diversity [22–25] and population dynamics [20–23,26], palaeoecology and niche partitioning [27,28], evolutionary trends through mechanisms like heterochrony and paedomorphosis [29–31] and growth dynamics along with physiological and reproductive drivers of ecological success [1,14,32–39]. Though these larger scale studies have been central to our increased comprehension of dinosaur evolution, they have also highlighted some pertinent caveats and necessary steps forward when working with data pertaining to dinosaur reproduction, growth and ontogeny. In this review, we summarize a few major areas that would benefit from targeted studies and revisions, and attempt to highlight ideal best practices and identify productive future directions for research.

## 2. Reproductive biology

Although many aspects of dinosaur reproduction have been explored, these have all relied on a highly uneven sampling of different dinosaur clades. Most eggs recovered have been associated with maniraptorifoms [40–44] (with limited information of other tetanurans [45,46]), hadrosaurids [13], one taxon of ceratopsid [47,48] and sauropodomorphs [49–54].

Late Cretaceous oviraptorosaurs have provided great insights into several reproductive factors [55]. For over a century, scientists hypothesized that dinosaurs laid hard-shelled eggs, like their closest living relatives (i.e. crocodilians and birds). In 1921, Roy Chapman Andrews discovered dinosaur nests containing elongated oval hard-shelled eggs in Mongolia. As *Protoceratops* was the most common dinosaur in the area, the taxon was hypothesized to be the egg layer. This led to the naming of *Oviraptor philoceratops*, the ‘egg thief’ assumed to be stealing *Protoceratops* eggs due to the unearthing of a skeleton next to a nest [56]. In 1993, the American Museum of Natural History uncovered *Citipati*, an oviraptorosaur brooding a clutch of elongated eggs, and changed the narrative of the previously thought scenario. The egg thief was in fact displaying parental care [57], a behaviour for which evidence has been reinforced by the discovery of seven clutch–adult associations [55]. In addition to parental care, paternal care specifically has been hypothesized in the group, based on clutch volumes and bone histology [58], along with open and exposed (or partially exposed) nests [41,59]. The latter is supported by various lines of evidence including the presence of a cuticle layer [59], and eggshell conductance (which depends on eggshell porosity) which is lower in oviraptor (and troodontid) eggs than in other non-avian dinosaurs [41,45,60]. Eggs with higher conductance (i.e. higher porosity) have been associated with high humidity and low oxygen conditions, suggesting buried eggs and underground development [61]. The evidence for paternal care and open nests was substantiated by the discovery that oviraptorosaurs had pigmented eggs [44].

Egg-laying amniotes have evolved many different egg and eggshell characteristics that offer various advantages for a species’ success. Crocodiles and turtles have unpigmented eggs that are protected by burial; some open-nesting birds have unpigmented eggs protected by continuous brooding [62], whereas others rely on egg pigmentation for protection. Bird eggs display the widest variety of size, shape and colour among modern vertebrates. Coloured eggs can allow for camouflage by blending in with the nesting background, protection against parasitism, antimicrobial effects, protection from solar radiation and eggshell mechanical reinforcement [44,62–67]. In 2017, protoporphyrin and biliverdin, the pigments responsible for the majority of colouration in extant bird eggs, were recovered using Raman microspectroscopy in the eggshells of various theropods [44]. This suggests that the Late Cretaceous oviraptorid *Heyuannia* and the dromaeosaur *Deinonychus* laid blue-green eggs, while some troodontids laid brown eggs and others laid speckled eggs. This entails that pigmented eggs had a single origin in eumaniraptorans much earlier than previously hypothesized, and that ornithischian and sauropodomorph eggs were unpigmented [35,44]. The blue-green egg colour in living birds has been associated with other reproductive behaviours, including paternal care [68] and communal nesting.

Communal nesting and nest fidelity have been previously hypothesized in sauropodomorph dinosaurs, including early branching members like *Mussaurus*, *Massospondylus* and *Qianlong shouhu*, as well as titanosaurs [54,69,70]. This is based on the vertical and lateral arrangement of several nests recovered from a single stratigraphic section, suggesting the seasonal return of multiple egg-laying females to a common site [49,71]. These extensive sauropodomorph nesting sites have also given insights into social behaviours in the group, such as age-segregated herds [49] and limited egg parental care [47]. The latter is based on the mechanical structure of these early branching sauropodomorph eggshells, which are believed to be soft, similar to the elusive *Protoceratops* eggs [47].

A clutch of eggs in Mongolia helped to shed light on the matter, as a dozen *Protoceratops* embryos were preserved in the fetal position, not surrounded by eggshells. With the help of Raman spectroscopy, and the mechanical properties of a broad sample of archosaurian eggs (relative thickness of eggshell membrane and crystalline layer), it was discovered that these *Protoceratops* embryos were in fact surrounded by mechanically soft eggshells. This condition was found to be ancestral for Archosauria, Ornithodira and Dinosauria [47]. Soft eggshells are more prone to dehydration and external stresses (such as a brooding parent) and were therefore likely buried in moist sediment, relying on external incubation and little parental care [47].

Most archosauromorphs (i.e. dinosaurs, birds, crocodilians and some turtles) lay hard-shelled eggs, defined as having a relatively thick calcareous layer organized in radially oriented prismatic structures (shell units); whereas most lepidosaurs (i.e. snakes and most lizards) lay soft-shelled eggs defined as having a relatively thin or absent calcareous layer but a relatively thick proteinaceous membrane organized into bundles of protein fibrils [72]. Some analyses include a third, intermediate category referred to as semi-rigid (i.e. leathery) eggs (i.e. tuataras, and some turtles and gekkos) [73]. The results and methods used when analysing the microstructure and mechanical properties of fossil eggshell have sparked some debate [54,73,74]. Several studies [74], including the 2024 analysis of the Chinese Early Jurassic sauropodomorph *Q. shouhu* eggs, suggest that the Raman spectroscopy signal pattern of organic matters detected in *Mussaurus* eggshells is not reliable evidence for mechanically soft-shelled eggs. This study points out that other characteristics, such as the eggshell thickness relative to egg mass, the rugose egg surface, the irregular egg shape and the eggshell fracturing pattern support the presence of a semi-rigid eggshell in *Q. shouhu* and other early branching sauropodomorphs (including *Mussaurus*) eggs, as well as in the first dinosaurs [54]. The *Q. shouhu* eggshells were also found to comprise a porous calcareous eggshell layer of approximately 160 μm thick (thicker than that of *Massospondylus carinatus* (80–100 μm)) with interlocking columnar eggshell units in the outer layer; and crown-shaped eggshell units with radially arranged calcite crystals in the inner layer. This microstructure is similar to what has been identified in *Lufengosaurus* eggs, potentially indicating that the latter only preserve the inner eggshell layer while the outer layer has weathered away. Similarly, the potential recrystallization of the eggshell in *M. carinatus* samples has been suggested to be problematic in the identification of columnar structural eggshell units [54,75]. The study of fossil eggshells and the macroevolution of eggshell structure is hindered by several factors. Firstly, taphonomic biases can affect results as the thicker calcareous layers are typically preserved, but the proteinaceous layers are not [36], and diagenesis can alter these layers. Secondly, there is a lack of congruence between definitions of eggshell types in the literature (i.e. hard, semi-rigid and soft) and phylogenetic tree calibration, suggesting that analyses of discrete characteristics (e.g. inner structures of shell units, pores and membrane elements) in a phylogenetic context should be focused on [73]. Finally, this may also be compounded by the fact that categories, such as hard, semi-rigid and soft, may be appropriate for defining eggshell structural diversity in extant taxa but not the broader diversity of extinct taxa.

Little is known about dinosaur incubation periods. In embryonic teeth, incremental lines of von Ebner that correspond to diurnal pulses of mineralization during odontogenesis can be seen [76,77]. These von Ebner lines have been used to estimate minimum incubation periods in ornithischian and theropod dinosaurs, revealing that both small (*Protoceratops*) and large taxa (*Hypacrosaurus*) had reptilian-like, slow incubation periods (estimated to be between 2.8 and 5.8 months) [76] whereas the theropod *Troodon formosus* appears to have a faster incubation period (estimated at 74 days) [42], intermediate between that of birds and reptiles. Dinosaur embryonic teeth have also been found to include several generations of teeth: null-generation teeth, simple conical teeth that get resorbed or shed during incubation, followed by functional teeth [78,79]. These multi-generational teeth are found in dinosaur relatives today, including geckos and alligators [80–82]; however, their phylogenetic distribution and function are poorly understood.

Fossilized dinosaur eggs have provided a wealth of information for reproductive biology studies. However, they remain rare and difficult to study. One of the limiting factors in sampling is the ability to confidently associate fossil eggs with the taxon that laid them. Unless fossil embryos are preserved *in ovo*, this association proves very challenging. Eggs are often referred to a taxon through adult–clutch associations (although this can also be problematic, as was demonstrated by the naming of *Oviraptor*), stratigraphic co-occurrence with abundant taxa (e.g. *Lourinhanosaurus* [45,46]) or through similarities in shape, size and eggshell microstructure to other previously identified taxa (e.g. titanosaur eggs from Brazil [83] and India [84]). Preserved embryos in eggs are extremely rare in the fossil record, although with the advances of micro-computed tomography (µCT), identifying and studying these have become more prevalent in recent years [52–54,78]. Despite being the most useful line of evidence for identifying egg layers, even embryos often cannot be confidently assigned to particular species. Many embryonic referrals have relied on biostratigraphic evidence, or morphological characters that are not necessarily diagnostic (except for *Mussaurus* embryos which show autapomorphies of the taxon [49]), or have been limited to the diagnosis to group level (e.g. oviraptorid oviraptorosaurs, titanosaur [52,53]). In order to confidently identify embryos, the morphological ontogenetic variation in taxa needs to be better understood (both extant and extinct), along with intraspecific variation (in both extant and extinct taxa, especially at the embryonic level). While much work has been done on modern reptilian taxa to better understand their embryonic ossification sequence [85–91], studies on their skeletal and dental morphological shape variation and growth have been limited. The same can be said of post-hatching ontogenetic morphological variation.

## 3. Osteohistological growth strategies

**Figure 1.**
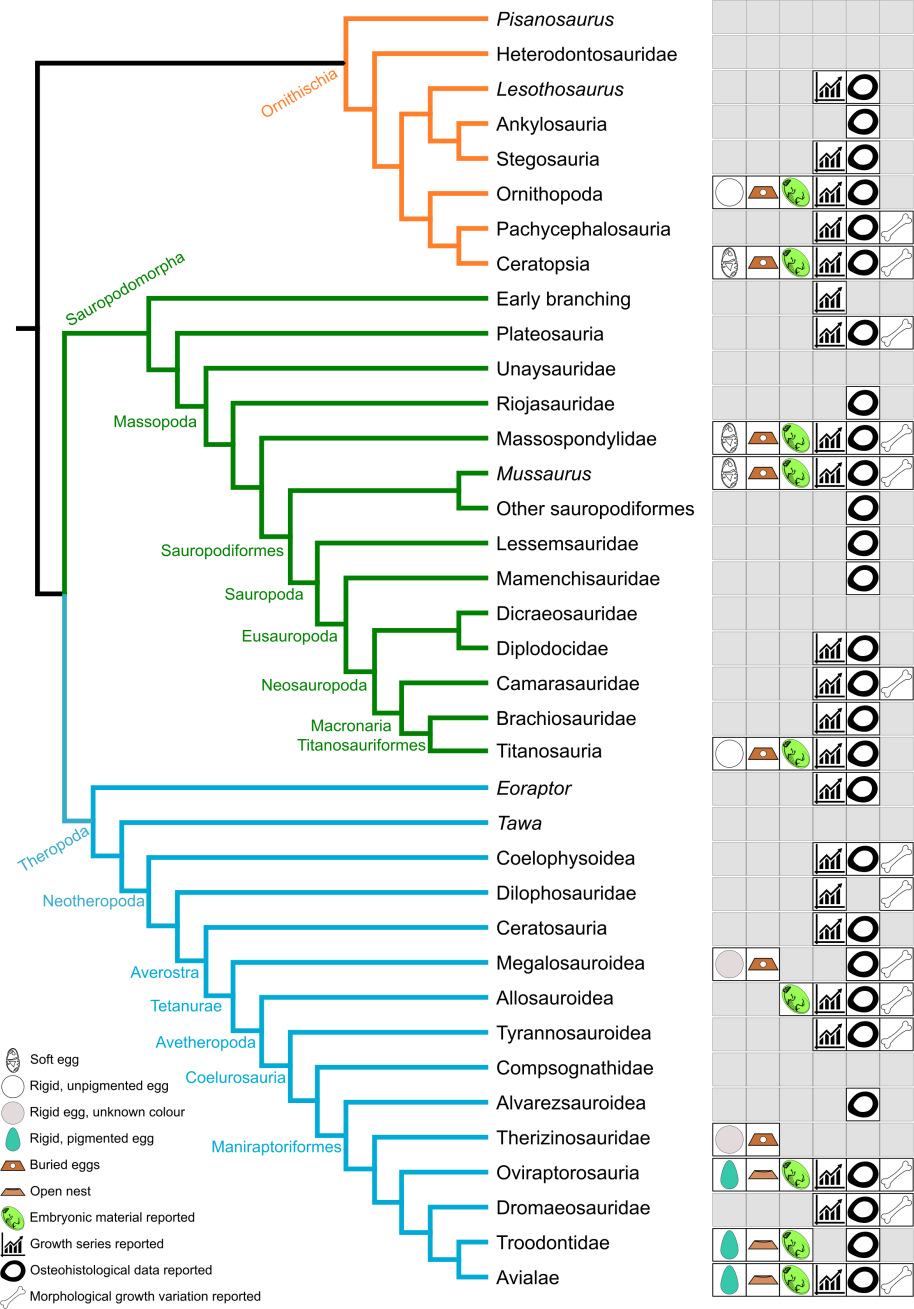
Simplified dinosaur phylogenetic tree showing selected ontogenetic data reported for each group (see electronic supplementary material for examples of relevant literature).

Palaeohistology is a crucial tool for investigating dinosaur growth and reproduction, and the most explored avenue of study (figure 1). By looking at bone and tooth microstructures (e.g. tissue types) and growth marks (e.g. lines of arrested growth, von Ebner growth lines, annuli, external fundamental systems), many hypotheses have been made regarding dinosaur growth rates and patterns, locomotory posture [92–94], ecological and physiological adaptations [95], as well as reproductive biology, including incubation period and sexual maturity [96]. Although traditional osteohistological methods require destructive sampling, µCT and synchrotron radiation micro-computed tomography (SrµCT) scanning offer potential avenues for non-destructive, high-fidelity maturity assessments [97]. Growth marks have proven to be particularly informative, allowing for the estimation of growth curves and determination of sexual maturity in taxa including *Massospondylus* [98], *Apatosaurus* [99], *Psittacosaurus* [37,100], *Maiasaura* [15], *Tenontosaurus* [101], *Allosaurus* [101], *Tyrannosaurus* [36,102–105] and *Shuvuuia* [37,106]. Using formulae derived from these growth curves, it has been assumed that a dinosaur’s age could be estimated using skeletal size.

Studies have, however, highlighted potential caveats with these correlations [107]. The presence of osteohistological growth variation is known in several taxa, including early-branching sauropodomorphs (*Plateosaurus* [108,109], *Massospondylus* [35,93], *Mussaurus* [94], *Antetonitrus* [110]), early-branching theropods (*Coelophysis* [34]), coelurosaurs (*Tyrannosaurus rex* [22]), early-branching Aves (*Confuciusornis sanctus* [111]) and non-iguanodontian ornithopod dinosaurs (*Jeholosaurus shangyuanensis* [112]). In most of these studies, this growth variation has been suggested to be linked to osteohistological growth plasticity, with growth being affected and determined by external environmental factors. This osteohistological growth variation has presented itself in several manners: differing growth strategies and tissue organization between specimens (e.g. cyclical and continuous growth recorded in *Mussaurus*), differing numbers of growth marks in specimens of similar sizes in a growth series (e.g. *Plateosaurus*, *Antetonitrus*, *Confuciusornis*), as well as irregular and unpredictable spacing between growth marks in an individual (*Massospondylus*, *Jeholosaurus*, *Coelophysis*, *Tyrannosaurus*). These three lines of evidence indicate a decoupling between size and ontogenetic age in many taxa; however, the first two highlight differences between individuals and are therefore more prone to confounding variables such as specimen identification. This decoupling between size and ontogenetic age could represent an early dinosaurian biological feature but could also be a reflection of relatively low sample sizes and a limited understanding of variation drivers in dinosaur osteohistological studies. To validate and reinforce these hypotheses, a better understanding of morphological growth variation in taxa is necessary (in extinct as well as extant taxa).

Interestingly, several examples of osteohistological growth plasticity are present in early branching members of most dinosaurian clades except for ornithischians, where the early taxon *Lesothosaurus* appears to have a predictable growth trajectory [113]. Although reported in extant vertebrates, such as mammals [114], crocodilians [115,116], lizards [117], amphibians [118] and birds [119], our understanding of osteohistological growth plasticity and the mechanisms that drive it are poorly understood. Testing this is logistically difficult, as it requires recorded data on environmental conditions (resources, diet and nutrition, temperatures), the ability to vary these conditions to better understand the triggers (wild versus captive, although the latter can prove to be problematic due to the unknown effects of breeding and genetic bottlenecking), and large sample sizes that include both growth series and several individuals of the same size.

Furthermore, while some research in living archosaurs has validated growth patterns observed using osteohistology in individuals of known ages [120], several studies have highlighted potential issues when using growth marks as indicators of age. One of the first, and by now well-known confounding factors when looking at osteohistological data is the fact that vertebrates display intraskeletal variation with different elements in a single skeleton showing different growth patterns (in tissue types and growth marks) [93,105,115,121–123]. This can make comparisons between taxa and between individuals difficult, especially in palaeontology, where researchers are restricted to fossils that are preserved. A more recent study looking at the relationship between growth marks and actual age in modern taxa (including reptiles, mammals and birds) found stark differences in growth mark counts when using petrographic ground sections versus stained microtomized sections [124]. Perhaps a more troubling result was that neither method unequivocally revealed the actual age of the individual specimens. While the study only looked at two limb bones (humerus and femur) in a single individual of nine species, it does emphasize the need for research on the methodological side of skeletochronology, especially when using growth marks.

## 4. Osteological morphological variation

Dinosaurs, like other vertebrates, did not simply increase in size during ontogeny but also underwent various osteological morphological changes throughout their life histories. These changes (e.g. cranial morphology, skeletal ‘robustness’, dentition, degree of ossification) can be dramatic and have even been the source of taxonomic debate—differentiating whether morphological differences between individuals derive from intraspecific ontogenetic variation or taxonomic diversity can be difficult, especially given the extreme ontogenetic trajectories many dinosaurs are hypothesized to have experienced (e.g. [125,126]). Therefore, constraining the kinds of changes that typified the ontogenies of dinosaurian subclades, and when in ontogeny these changes occurred, is a major research goal. Although some suggested ontogenetic changes in dinosaurs have no clear modern analogues (e.g. ceratopsian frill morphology [127]), certain skeletal changes, such as the production of ossified muscle attachments and sutural fusion events, are irreversible and occur in the ontogenies of most living reptiles (e.g. [128–131]), so similar features are likely to be ontogenetically variable in extinct dinosaurs [21]. However, at what stages these changes might occur during ontogeny is unknown for most dinosaurian subclades. For example, a sutural fusion could indicate that a given individual has gone through this ontogenetic change, and this is therefore informative as to the relative maturity of this individual. However, without constraints on when in ontogeny this change typically occurs, and how this ontogenetic change has evolved through time, there is little broader context that could be given for this character state. Because most dinosaurian taxa are represented by few individuals, it is crucial to use exemplar taxa with well-represented ontogenetic series (e.g. *Massospondylus* [35,93], *Coelophysis* [34], *Allosaurus* [132], *Confuciusornis* [111], *Psittacosaurus* [92], *Maiasaura* [16]) as ‘model organisms’ to help interpret ontogenetic changes across Dinosauria. Constraining putative ontogenetic changes is especially important when attempting to use morphological evidence to assess maturity status or when phylogenetically informative character states are ontogenetically variable [21].

Similar to osteohistological signals of growth (see above), the sequence of morphological changes that occurs during ontogeny can also vary intraspecifically (i.e. sequence polymorphism [133]). Different individuals of the same species, and even the same population, can develop in different ways—the sequence of morphological changes may differ, body size can vary widely between individuals of the same maturity stage and some ontogenetically variable features may not necessarily be present in all individuals. High intraspecific variation in discrete ontogenetic character states has been suggested to be especially prevalent among the earliest diverging dinosaurs and their closest relatives [134–136], but intraspecific variation in putatively ontogenetic morphology has been reported in many taxa (e.g. *Masiakasaurus* [137], *Maiasaura* [138] and *Psittacosaurus* [139]). How this intraspecific morphological variation may relate to osteohistological variation is unclear, but the two signals appear to be disjunct: several taxa have been reported to exhibit high intraspecific variation in osteohistological signal while lacking comparatively high variation in morphology (e.g. *Allosaurus* [96,134]; *Massospondylus* [93]). Combined osteohistological and morphological studies [34] from exemplar taxa spanning the major dinosaurian clades are necessary to untangle this signal.

## 5. Ontogeny and macroevolution

Parallels between dinosaur ontogenetic and macroevolutionary patterns have long been suggested [140,141], through mechanisms like heterochrony (including paedomorphosis, peramorphosis and recapitulation). Both the sauropod and bird skull shapes have been attributed to evolution acting along ontogenetic trajectories. Both have been suggested to have evolved through paedomorphosis [30,79,142], although the sauropod skull has also been attributed to a predisplacement-type shift in developmental timing from the ancestral anchisaurian condition [29], and several aspects of the avian skull have been suggested to have arisen via peramorphosis [143]. Locomotory shifts between bipedalism and quadrupedalism occurred several times during the evolution of ornithischians and sauropodomorphs, and have also been hypothesized to be linked to ontogenetic locomotory shifts [31,50].

Osteohistological growth data have also been used to investigate macroevolutionary patterns in archosaur lineages, such as gigantism in sauropod dinosaurs [33,39]. The latter are well known for being the largest land animals to have roamed the earth (weighing between 10 and 70 tonnes). Osteohistological data show that while accelerated, uninterrupted growth and a lack of developmental plasticity are traits that evolved near the origin of sauropod gigantism; accelerated, seasonally interrupted growth was already present in smaller (1−2 tonnes) non-sauropodan sauropodomorphs [39]. This suggests that accelerated growth may have been a prerequisite for the evolution of gigantism in the clade, but did not evolve for it specifically. Among non-avian theropods, changes in rate and timing of growth (e.g. truncation, prolongation, acceleration), as revealed by osteohistology, have been suggested to be an important mechanism by which body size has evolved, including trends in both gigantism and miniaturization [144]. These types of macroevolutionary mechanisms are difficult to test and require both relatively complete and well-established ontogenetic and phylogenetic samples. Specimens included need to have confirmed taxonomic identities, their ontogenetic maturity needs to be known and phylogenetic relationships should be resolved. These factors are hindered by several obstacles, including the difficulty to differentiate intraspecific morphological variation from interspecific morphological variation and ontogenetic variation.

## 6. Concluding remarks and recommendations for future work

### (a) Fieldwork and exhaustive use of pre-existing fossil collections

Sampling is a well-known issue in palaeontology, with researchers being restricted to whichever rare fossils are preserved. This impacts sample sizes, phylogenetic sampling and ontogenetic studies, among others. Fossil collecting efforts focusing on increasing ontogenetic sample sizes should include all taxonomically informative specimens, even partial skeletons and isolated elements, as these could eventually result in valuable data points for studies on growth and development. While unbiased continued fieldwork is a much-needed way of increasing sampling, there is also great value in revisiting existing fossil collections, as well as capitalizing on known, already accessioned specimens. Building out the big picture of dinosaur reproduction and growth requires multiple specimens of individual taxa and, if possible, growth series, to be scrutinized using a multidisciplinary approach. These include traditional methods such as qualitative observations of morphological variation (both cranial and postcranial), along with more complex data collection (such as µCT scanning, Raman spectroscopy, osteohistology). Several published ontogenetic series of dinosaurs have yet to be studied to their full potential (as is evidenced by the knowledge gaps in figure 1), and there is undoubtedly a wealth of fossils that remain unpublished in need of reassessment.

### (b) Methodical collection and reposition of µCT scan data

The use of µCT and SrµCT scanning allows for a wide variety of data to be collected from specimens in a non-destructive manner (including but not restricted to visualization of obstructed morphology as well as small specimens like embryos, quantitative morphological data, osteohistological data, dental replacement and histological data, and internal physiological structures like pneumaticity). The generation of digital scans allows for improved sampling (of both extant and extinct taxa), with easy access to and exchange of data between researchers, and in some instances offers the only opportunity to compile large and broad-scaled datasets in a non-destructive manner. Databases like the openVertebrate project (oVert) are an incredibly beneficial initiative. These types of systematic collecting and repositing of scan data from museum collections, along with the increased demand from journals for raw µCT scan data to be accessioned on open access platforms like Morphosource and Phenome10k upon publication, allow for an abundance of both focused and broad-scaled research projects in the future.

### (c) Assessing ontogenetic maturity

Taking ontogenetic maturity of fossil specimens into consideration is non-negotiable when investigating almost any aspect within an evolutionary, taxonomic or palaeobiological framework. Ontogenetic maturity has a direct impact on morphology (both macro and micro), which is the most fundamental level of data in our field, having a cascading direct effect on taxonomic identification, phylogenetics, population structure and palaeoecology, life-history strategies, functional anatomy and macroevolutionary trends and mechanisms (like heterochrony). Including ontogenetic maturity discussions when describing specimens, or naming new taxa provides explicit stipulations that can then be considered when including the said specimen/taxon in datasets. Although assessing ontogenetic maturity has been done using a wide variety of methods (e.g. suture fusion levels, osteohistological analyses), these criteria vary depending on which saurian clade is being investigated [21]. It is therefore recommended that a combination of different methods be used and that clade-specific ontogenetic maturity criteria be followed. The use of µCT scanning and SrµCT scanning has proven to be a useful tool for assessing ontogenetic maturity in a detailed and non-destructive manner [97,145].

### (d) Establishing consistency in terminology and methods

The field of palaeontology today is more productive than ever. As dataset sizes increase, and technologies advance, new research questions and methods for testing these arise. While this increase in productivity aids in building a bigger picture of Earth’s history, it can also contribute to complications. This is noticeably true with the increased inconsistency in terminologies used and methods of data collection (even as simple as where measurements are taken, or how phylogenetic characters are interpreted). For example, Griffin *et al*. [26] illustrated the various and inconsistent terms used when assessing and describing maturity stages (e.g. ‘juvenile’, ‘subadult’, ‘adult’, etc.) without consistent meaning across the literature. Similarly, Legendre *et al*. [73] highlighted the issues arising from the incongruence in definitions and methods used when studying ‘soft’, ‘hard’ and ‘semi-rigid’ reptilian eggshells. Both comprehensive studies provide guidelines for overcoming some of these hurdles. These irregularities can lead to subjective interpretations, inter-scientist errors and variation and, ultimately, cross-study incoherence. While ‘gold standards’ are unrealistic, several steps can be taken to standardize the literature, allowing for better clarity and consistency. These include: describing and illustrating methods in the most unambiguous way possible to reduce subjective interpretation (e.g. illustrating where measurements were taken, illustrating data matrices to show how character states were interpreted, using quantitative descriptors and criteria when possible); using consistent and latest terminology and explicitly stating and referencing these; describing any and all modifications that are applied to reused datasets, methods and terminologies.

### (e) Building extant comparative datasets

Palaeontology is restricted by many factors, including, but not limited to, sample sizes, preservational biases, geographical biases and collection biases. Another confounding factor is the availability and understanding of modern comparative analogues. Extant ecosystems provide the best, and only opportunities to explore and interpret findings from the fossil record. While past dinosaur lineages do not have ideal direct comparatives available (for example, sauropodomorphs and ornithischians have no direct descendants, and animals alive today do not reach the same sizes or morphological and functional diversity), there are many living groups of vertebrates that present ideal study systems.

As aforementioned, osteological morphological data are the most fundamental baseline of data in our field, providing insights into many aspects of biology and palaeontology. Detailed osteological monographic descriptions of modern taxa are available for relevant lineages such as squamates [146–148] and some birds [149,150]; however, despite the importance of morphological data in biological research, these are very limited and often aimed at veterinary practices [151,152]. Our understanding of osteological morphological intraspecific, interspecific and ontogenetic variation is very limited in extant vertebrates. This is, in part, due to the fact that modern species taxonomy and phylogenetic relationships can rely on information that is not available in fossils, such as integument and molecular data.

The relationship between osteological morphological variability and osteohistological variability is even less well explored in living vertebrates. The latter provides the best opportunity to observe the relationships between hard tissues and every aspect of vertebrate biology, behaviour and other natural and life-history traits. These observations can then be applied to the fossil record to make palaeoecological and palaeobiological inferences. Osteohistological studies in extant dinosaur relatives are becoming more numerous, but they remain scarce overall. In crocodylomorphs, these are phylogenetically narrow with the majority of studies focusing on alligators and a few on caimans, possibly due to the fact that specimens for these are easily accessible in captivity [115,153–155]. Furthermore, these studies typically include specimens obtained from a single refuge and are unlikely to represent the range of growth variation in a given taxon across its range, and the effect of varying environmental conditions on ontogeny. Birds being the only living descendants of dinosaurs present an ideal comparative dataset [156–158]; however, their ontogenetic patterns can be difficult to study due to their rapid growth rates. Several recent studies have reported a decoupling of observed growth marks in specimens and the actual age of the individual [124,157,158]. This has major implications for our interpretation of physiology and growth histories in extinct taxa.

Concerted efforts need to be made by palaeontologists and palaeobiologists to better understand living animals, and ground truth methods in extant lineages, before being able to make inferences about extinct groups. This would allow for the establishment of a solid foundation to interpret the complex history of dinosaurs. These studies should explore phylogenetically and ontogenetically broad extant vertebrate samples, and take into account how the conditions in which the specimens were living (i.e. captive versus wild) may affect the questions being asked, as well as the results (such as through genetic bottlenecking, selective breeding, diets, substrate, environmental conditions, etc.).

## Data Availability

Supplementary material is available online [159].
